# External Validation of a Predictive Model for Acute Skin Radiation Toxicity in the REQUITE Breast Cohort

**DOI:** 10.3389/fonc.2020.575909

**Published:** 2020-10-30

**Authors:** Tim Rattay, Petra Seibold, Miguel E. Aguado-Barrera, Manuel Altabas, David Azria, Gillian C. Barnett, Renée Bultijnck, Jenny Chang-Claude, Ananya Choudhury, Charlotte E. Coles, Alison M. Dunning, Rebecca M. Elliott, Marie-Pierre Farcy Jacquet, Sara Gutiérrez-Enríquez, Kerstie Johnson, Anusha Müller, Giselle Post, Tiziana Rancati, Victoria Reyes, Barry S. Rosenstein, Dirk De Ruysscher, Maria C. de Santis, Elena Sperk, Hilary Stobart, R. Paul Symonds, Begoña Taboada-Valladares, Ana Vega, Liv Veldeman, Adam J. Webb, Catharine M. West, Riccardo Valdagni, Christopher J. Talbot, REQUITE consortium, Yolande Lievens

**Affiliations:** ^1^Cancer Research Centre, University of Leicester, Leicester, United Kingdom; ^2^Division of Cancer Epidemiology, German Cancer Research Center (DKFZ), Heidelberg, Germany; ^3^Fundación Pública Galega Medicina Xenómica, Santiago de Compostela, Spain; ^4^Instituto de Investigación Sanitaria de Santiago de Compostela, Santiago de Compostela, Spain; ^5^Radiation Oncology Department, Vall d'Hebron Hospital Universitari, Vall d'Hebron Barcelona Hospital Campus, Barcelona, Spain; ^6^Fédération Universitaire d'Oncologie Radiothérapie d'Occitanie Méditérranée, Département d'Oncologie Radiothérapie, ICM Montpellier, INSERM U1194 IRCM, University of Montpellier, Montpellier, France; ^7^Department of Oncology, University of Cambridge, Cambridge, United Kingdom; ^8^Department of Human Structure and Repair, Ghent University, Ghent, Belgium; ^9^Department of Radiation Oncology, Ghent University Hospital, Ghent, Belgium; ^10^University Cancer Center Hamburg, University Medical Center Hamburg-Eppendorf, Hamburg, Germany; ^11^Translational Radiobiology Group, Division of Cancer Sciences, University of Manchester, Manchester Academic Health Science Centre, The Christie NHS Foundation Trust, Manchester, United Kingdom; ^12^Centre for Cancer Genetic Epidemiology, Department of Oncology, Strangeways Research Laboratory, University of Cambridge, Cambridge, United Kingdom; ^13^Fédération Universitaire d'Oncologie Radiothérapie d'Occitanie Méditérranée, Département d'Oncologie Radiothérapie, CHU Carémeau, Nîmes, France; ^14^Hereditary Cancer Genetics Group, Vall d'Hebron Institute of Oncology (VHIO), Vall d'Hebron Hospital Campus, Barcelona, Spain; ^15^Prostate Cancer Program, Fondazione IRCCS Istituto Nazionale dei Tumori, Milan, Italy; ^16^Department of Radiation Oncology, Department of Genetics and Genomic Sciences, Icahn School of Medicine at Mount Sinai, New York, NY, United States; ^17^MAASTRO Clinic, GROW School for Oncology and Developmental Biology, Maastricht University Medical Center, Maastricht, Netherlands; ^18^Department of Radiation Oncology, University Hospitals Leuven/KU Leuven, Leuven, Belgium; ^19^Department of Radiation Oncology 1, Fondazione IRCCS Istituto Nazionale dei Tumori, Milan, Italy; ^20^Department of Radiation Oncology, Universitätsklinikum Mannheim, Medical Faculty Mannheim, Heidelberg University, Mannheim, Germany; ^21^Independent Cancer Patients' Voice, London, United Kingdom; ^22^Department of Radiation Oncology, Complexo Hospitalario Universitario de Santiago, Servizo Galego de Saúde (SERGAS), Santiago de Compostela, Spain; ^23^Department of Genetics and Genome Biology, University of Leicester, Leicester, United Kingdom; ^24^Department of Hematology and Hemato-Oncology, Università degli Studi di Milano, Milan, Italy

**Keywords:** validation, prediction model, early toxicity, radiotherapy, breast cancer

## Abstract

**Background:** Acute skin toxicity is a common and usually transient side-effect of breast radiotherapy although, if sufficiently severe, it can affect breast cosmesis, aftercare costs and the patient's quality-of-life. The aim of this study was to develop predictive models for acute skin toxicity using published risk factors and externally validate the models in patients recruited into the prospective multi-center REQUITE (validating pREdictive models and biomarkers of radiotherapy toxicity to reduce side-effects and improve QUalITy of lifE in cancer survivors) study.

**Methods:** Patient and treatment-related risk factors significantly associated with acute breast radiation toxicity on multivariate analysis were identified in the literature. These predictors were used to develop risk models for acute erythema and acute desquamation (skin loss) in three Radiogenomics Consortium cohorts of patients treated by breast-conserving surgery and whole breast external beam radiotherapy (*n* = 2,031). The models were externally validated in the REQUITE breast cancer cohort (*n* = 2,057).

**Results:** The final risk model for acute erythema included BMI, breast size, hypo-fractionation, boost, tamoxifen use and smoking status. This model was validated in REQUITE with moderate discrimination (AUC 0.65), calibration and agreement between predicted and observed toxicity (Brier score 0.17). The risk model for acute desquamation, excluding the predictor tamoxifen use, failed to validate in the REQUITE cohort.

**Conclusions:** While most published prediction research in the field has focused on model development, this study reports successful external validation of a predictive model using clinical risk factors for acute erythema following radiotherapy after breast-conserving surgery. This model retained discriminatory power but will benefit from further re-calibration. A similar model to predict acute desquamation failed to validate in the REQUITE cohort. Future improvements and more accurate predictions are expected through the addition of genetic markers and application of other modeling and machine learning techniques.

## Introduction

Survivorship issues and quality-of-life (QoL) are becoming an increasingly important research focus in cancer care (1). Breast cancer survival has improved markedly, with current predicted 10-year survival rates in excess of 80% (2). Over 70% of breast cancer patients undergo radiotherapy, usually in the adjuvant setting following surgery. Radiotherapy reduces the risk of local recurrence and contributes to a reduction in overall mortality (3). Nevertheless, breast radiotherapy can be associated with several side-effects (toxicity). Acute (or early) toxicity includes breast erythema (reddening) and desquamation (skin loss) and occurs within 90 days of treatment (4). While late side-effects of radiotherapy are concerning due to their potential irreversibility, acute toxicity may cause considerable patient morbidity and can have adverse effects on the cosmetic outcome from oncoplastic breast surgery and reconstruction (5, 6). There is some evidence that if sufficiently severe, early toxicity can be associated with clinically significant late toxicity (7). Invariably, surgeons' treatment recommendations are influenced by their perception of potential adjuvant treatment complications such as from radiotherapy (8, 9). Nevertheless, there is considerable variation between individual patients' normal tissue reaction to radiotherapy. Being able to stratify individual patients according to their risk of radiation toxicity would enable breast surgeons to take this information into account when advising patients about the risks and benefits of different surgical treatment options, or even suggest a change to the sequence of surgery and adjuvant treatment including radiotherapy (10).

In the field of medical physics, many radiobiological predictive models have been proposed with the aim of preserving normal tissue, mostly focused on late toxicity. Normal tissue complication probability (NTCP) models, such as the Lyman-Kutcher-Burman (LKB) model, incorporate the linear quadratic (LQ) model of cell killing (11, 12). Many of these dosimetric models have already been integrated into radiotherapy treatment planning systems. They generally take the form of simplified empirical models consisting of dose distribution parameters, and the risk of toxicity is assumed to depend on the mean dose to the respective target organ or the amount of damaged tissue (13).

In prostate radiotherapy, it has been shown that dosimetric models for late rectal toxicity can be improved by including clinical and other treatment risk factors, such as prior abdominal surgery, colorectal disease and diabetes (14, 15). In breast radiotherapy, several studies have investigated the association of clinical and treatment risk factors with acute skin toxicity, although none have reported a clinical prediction model as such (16–25). Integrated clinical prediction models capable of identifying patients at risk of clinically significant side-effects have now been developed in different disease sites, the majority predicting late toxicity with moderate performance (AUC ranging from 0.60 to 0.75) (26–28). There are also an increasing number of published models predicting acute toxicity, although none for breast radiotherapy (29–31).

For surgeons and other clinicians, models that include common clinical and treatment predictors are of particular interest because this obviates the need for detailed patient dosimetry and dose-volume histograms from radiotherapy planning scans. It would allow clinicians to estimate toxicity risk at the time of breast cancer diagnosis and before any treatment is planned. In breast reconstruction surgery, a small number of clinical risk models for various 30-day complications have been published (32, 33), of which some have been validated for select endpoints (34, 35). However, these models are chiefly designed to predict surgical side-effects, such as implant loss, surgical site infection and seroma, and include radiotherapy as a binary predictor variable only.

In the absence of an available prediction model in the literature, the aim of this study was to develop and externally validate predictive models for acute breast radiation toxicity in the REQUITE study breast cohort using published clinical and treatment predictors of acute skin toxicity in the REQUITE study breast cohort.

## Methods

This study was designed using data from patients who underwent breast-conserving surgery (BCS) and adjuvant external beam radiotherapy (EBRT) enrolled in three Radiogenomics Consortium (RGC) studies and the REQUITE cohort study. Candidate variables associated with acute breast radiation toxicity were identified from the existing literature. In the absence of predictive models in the literature, predictive models for acute radiation toxicity endpoints were first developed in combined RGC patient cohorts, then validated in the REQUITE patient cohort. This was a TRIPOD type 3 study, representing model development and validation using a separate dataset (36).

### Model Development Cohorts

The German ISE cohort (16) included 478 breast cancer patients treated with conventional 3D conformal whole breast EBRT plus either photon or electron tumor bed boost (except for 19 patients) recruited into a prospective patient cohort at four centers in Southwest Germany between 1998 and 2001, with documented acute radiotherapy toxicity at baseline, at cumulative doses of 36–42 Gy and 44–50 Gy, at the end of radiotherapy, and 6 weeks following radiotherapy. None of the patients in ISE received chemotherapy. All patients from the ISE cohort were included in this study. The ISE study was approved by the Ethical Committee at the University of Heidelberg, Germany (reference No. 37/98).

The LeND cohort (37) consists of 633 breast cancer patients treated with conventional 3D conformal whole breast EBRT using tangential fields and documented normal tissue toxicity recruited at varying time points (up to several years) after breast radiotherapy ± boost in Leicester, Nottingham and Derby (UK) between 2008 and 2010. Acute toxicity was collected from medical records. After excluding the first 154 patients without data on acute toxicity, and 119 patients who had chest wall radiotherapy following mastectomy, 390 patients treated with EBRT following BCS from the LeND cohort were included in this study. The LeND study was approved by the Research Ethics Committee (reference no. 08/H0405/57).

The Cambridge cohort (19) comprised 1,144 women who received adjuvant whole breast EBRT following BCS as part of the Cambridge IMRT trial (UK) following the standard hypo-fractionated regimen (40 Gy in 15 fractions), 411 of whom were randomized to manual forward-planned intensity-modulated radiotherapy (IMRT) to improve dose homogeneity (reduce the volumes receiving >107 and <95% of the prescribed dose) in the irradiated breast. The remainder of patients were treated with 3D-conformal radiotherapy using wedged tangential fields. Toxicity was documented weekly during treatment according to the RTOG scale. All patients from the Cambridge cohort were included. The study was approved by the Cambridge Research Ethics Committee and written consent was obtained from all patients to use their data for research purposes.

### Validation Cohort

The multicenter REQUITE breast cancer patient cohort was recruited prospectively in seven European countries and the USA between 2014 and 2016. The REQUITE study was conceived as an international multicenter validation cohort for predictive models of radiation toxicity with standardized prospective data collection (38). Patient baseline characteristics and methodology have been described in detail elsewhere (39). All 2,057 enrolled patients were treated with BCS followed by EBRT according to local protocol, approximately half of whom were treated with IMRT, with a lower proportion in France and no IMRT at Italian or US centers. The majority patients received a tumor-bed boost (64%), ranging from <20% at the French, Italian and Spanish centers to over 80% at the Belgian center, given either simultaneously (*n* = 257) or sequentially (*n* = 1,138). Patients with invasive breast cancer in Belgium and the UK were treated using the START-B hypofractionated regimen. Although late toxicity was the main endpoint in REQUITE, data collected at the end of radiation treatment was used to document acute toxicity. All patients gave written informed consent. The study was approved by local ethics committees in participating countries (UK NRES Approval 14/NW/0035) and registered at http://www.controlled-trials.com (ISRCTN98496463). Characteristics of all cohorts included in this study are summarized in Table 1.

**Table 1:** Summary study characteristics of eligible patients from the three RGC derivation cohorts and the REQUITE validation cohort (RT, radiotherapy).

	**LeND**	**ISE**	**Cambridge**	**REQUITE**
Total patients in cohort (*n*)	663	478	1144	4438
Eligible patients (BCS+EBRT)	409	478	1,144	2,057
Location	Leicester, Nottingham, Derby (UK)	SW Germany	Cambridge (UK)	Western Europe, United States
Study design	Retrospective	Prospective	Prospective	Prospective
Recruitment year (range)	2008–2010	1998–2001	2003–2007	2014–2016
Treatment year (range)	1998–2008	1998–2001	2003–2007	2014–2016
Toxicity assessment scale	RTOG	Modified CTCAE v2	RTOG	CTCAE v4
Toxicity assessment time points	(From records) end-of-RT	Start-of-RT cumulative 36–42 Gy cumulative 44–50 Gy end-of-RT 6 weeks after RT	Weekly during RT end-of RT	Start-of-RT end-of-RT
Age (median, range)	59 (33–87)	61 (27–87)	59 (26–84)	58 (23–90)
Whole breast dose (Gy, median, range)	50 (40–50)	50 (44–56)	40	50 (28.5–56)
Whole breast fractions (median, range)	25 (11–25)	25 (22–29)	15	25 (5–31)
Boost (proportion of patients)	10%	90%	65%	64%
Toxicity scale used	RTOG	CTCAE v2.0	RTOG	CTCAE v4.0
BMI ≥25 (proportion)	66%	48%	63%	54%
Smoker (current or previous)	13%	30%	15%	43%
Chemotherapy	28%	None	20%	30%
Diabetes	8%	6%	5%	6%
Hypertension	35%	32%	Not available	28%
Cardiovascular disease	6%	16%	10%	7%
Tamoxifen use	75%	80%	66%	76%

### Endpoint Definition

Radiation toxicity in REQUITE was scored using CTCAE (Common Terminology Criteria for Adverse Events; Table 2) v4.0 (40). CTCAE v4.0 has separate scales for radiation dermatitis (erythema) and skin ulceration (desquamation), both of which are relevant to the acute response to radiotherapy in the breast. For both LeND and Cambridge cohorts, acute skin toxicity was scored according to the RTOG (Radiation Therapy Oncology Group; Table 2) scale, which is mostly based on target organ or body region (e.g., larynx, upper GI, skin) (41). The German ISE study used a modified version of the Common Toxicity Criteria (CTCAE v2.0) scale for erythema, where grade 2 was subdivided into three sub-grades, with 2c being defined as ≥1 moist desquamation or interruption of treatment due to side-effects and grade 2a and 2b comprising moderate and brisk erythema, respectively.

**Table 2:** RTOG and CTCAE v4.0 toxicity scales for acute skin reaction and ulceration.

**Toxicity**	**Grade 1**	**2a**	**2b**	**3**	**4**
RTOG Skin	Follicular, faint or dull erythema/epilation/dry desquamation/decreased sweating	Tender or bright erythema ± dry desquamation	Patchy moist desquamation; moderate oedema	Confluent, moist desquamation other than skin folds, pitting edema	Ulceration, hemorrhage, necrosis
CTCAE v4.0 Radiation dermatitis	Faint erythema or dry desquamation	Moderate to brisk erythema or patchy moist desquamation (2c^*^), mostly confined to skin folds and creases; moderate edema (tenderness is graded separately in the Pain category)		Confluent moist desquamation ≥1.5 cm diameter and not confined to skin folds; pitting edema	Skin necrosis or ulceration of full thickness dermis; may include bleeding not induced by minor trauma or abrasion
CTCAE v4.0 Skin ulceration	Combined area of ulcers <1 cm; non-blanchable erythema of intact skin with associated warmth or oedema	Combined area of ulcers 1–2 cm; partial thickness skin loss involving skin or subcutaneous fat		Combined area of ulcers >2 cm; full-thickness skin loss involving damage to or necrosis of subcutaneous tissue that may extend down to fascia	Any size ulcer with extensive destruction, tissue necrosis, or damage to muscle, bone, or supporting structures with or without full thickness skin loss

* *Sub-scales of CTCAE v2.0 used in the ISE study*.

This raised the issue of how to deal with the use of different toxicity scales and assessment time points in the previously assembled cohorts and the REQUITE validation cohort. Where multiple measurements were available, maximum recorded toxicity was used. To ensure comparability with previous studies, the following endpoints were considered where they occurred within 90 days of the start of treatment (acute toxicity) according to the different grading systems:

a) Acute erythema: RTOG or CTCAE grade≥2 (at least moderate to brisk erythema);
b) Acute desquamation: RTOG grade≥2b (patchy moist desquamation) or CTCAE grade ≥2c erythema (moist desquamation) or CTCAE grade≥1 skin ulceration, implying that skin integrity has been broken, either over the breast or in the infra-mammary fold.

### Selection and Definition of Candidate Predictors

The literature was searched through Medline using the MeSH keywords “radiation injury,” “breast neoplasm,” “radiotherapy,” “radiation tolerance,” and “risk factors,” and through PubMed using keywords “radiation injury,” “normal radiation toxicity,” “acute,” “radiotherapy,” “breast cancer,” “radiosensitivity” and “risk factor” or “predictor” or “radiogenomics.” Reference lists from identified papers or review articles were also searched. Candidate predictor variables in the literature were considered for validation where their association with acute breast radiation toxicity endpoints on multivariate analysis was reported in at least one publication.

To ensure comparability of measures of breast size, such as breast diameter or bra size, these were converted to a single continuous variable for the purpose of this study by adding bra cup and band sizes, to represent “sister” sizes equal to the same breast volume (according to http://www.sizechart.com/brasize/sistersize/index.html). For instance, a UK size 34B bra holds an approximate breast volume equal to 32C, ~390 cc. In the Cambridge trial cohort, breast size was graded as a categorical variable and converted accordingly.

For each patient in the REQUITE and the other three RGC cohorts, information comprising candidate predictor variables and relevant study endpoints were extracted from the data. Hypertension was not recorded in the Cambridge trial cohort, and post-operative infection was not available in the LeND and ISE cohorts. Observations on body mass index (BMI) and breast size were missing from 22 and 17% of patients, respectively, in the three combined RGC cohorts, while information on the remaining candidate predictor variables was missing in between 0.5 and 3% of patients across all cohorts.

### Statistical Methods

Both endpoints were considered as dichotomized (binary) outcome measures. Where a patient had multiple measures of acute toxicity within the specified time period, the maximum grade of toxicity recorded was used. Cases with high baseline toxicity defined as grade≥2 were excluded from the analysis. Statistical analyses were carried out in Stata™ version 15.1. Continuous variables are presented as medians (with ranges), and categorical/binary variables as counts and percentages.

In order to minimize bias from analyzing only complete cases, multiple imputation (MI) was used to replace missing values by means of a chained equation approach based on all candidate predictors excluding hypertension (42). Ten imputed datasets were created for missing variables and then combined across all datasets using Rubin's rule to obtain final estimates (43). The number of imputations (*m* = 10) was determined by the percentage of incomplete observations per variable to reduce the error associated with estimating the regression coefficients, standard errors and the resulting *p*-values (44). On the basis of an estimated 900 cases of acute erythema and 175 cases of acute desquamation in the three combined RGC cohorts, the consideration of nine candidate predictor variables in this analysis satisfied the methodological constraint of at least 10 events per variable (EPV) required to reduce issues with over-fitting in predictive modeling (45).

To develop clinical prediction models, a generalized linear mixed model (GLMM, *xtlogit*) was fitted in the original dataset combining three RGC derivation cohorts to model the probability of each toxicity endpoint. GLMMs are an extension of mixed models and generalized linear models (GLMs) to allow for inclusion of both fixed and random effects across different study cohorts or cohorts enrolling at multiple centers. Like GLMs, a link function is applied, such as the *logit* link. Initially, a full model comprising all included predictor variables was fitted, followed by stepwise backwards elimination to select the candidate variables to include in the final prediction model (with *p* < 0.1 taken conservatively to warrant inclusion). After elimination, each excluded predictor was re-inserted into the final model to further check whether they became statistically significant at this stage.

The equation for the log odds for each acute breast endpoint was formed using the estimated β coefficients multiplied by the predictors included in the model together with the intercept across cohorts. The predicted risk of toxicity can thus be calculated:

$$\begin{array}{l} predicted\;risk=\frac{e^{\log\;odds}}{1+e^{log\;odds}} \end{array}$$

Discrimination of the fitted models was assessed by calculating the c-statistic (AUC from the logistic model, plotting sensitivity over 1-specificity) and examining the calibration plot across tenths of predicted risk. A c-statistic of 1 indicates perfect discrimination, whereas 0.5 indicates no discrimination. A calibration slope of 1 indicates perfect calibration and would be expected across the original datasets as the model is being developed in the same data (apparent performance).

To control for optimism (over-fitting), the model development process was repeated in 100 bootstrap samples. Each model was applied to the same bootstrap sample to quantify apparent performance, and then to the original dataset to evaluate test performance (c-statistic and calibration slope) and optimism (difference in test performance and apparent performance). To estimate overall optimism, the average calibration slope across all bootstrap samples was calculated and multiplied as a shrinkage coefficient by each variable's β coefficient and the intercept of the model derived in the original dataset to produce a final model for each toxicity endpoint.

The final models were applied to patients in the REQUITE validation cohort to predict the log odds of acute erythema or acute desquamation based on the presence or absence of one or more of the predictor variables. In this external validation step, the intercept of each final model was re-calibrated by subtracting the estimated intercept of the model in the REQUITE validation cohort. Performance of the model in the validation cohort was again assessed by calculating the c-statistic (AUC) and examining the calibration plot across tenths of predicted risk. Overall accuracy was measured by calculating a Brier score, which is the sum of mean square errors between predicted risk and observed outcome for each patient, with a zero score indicating total accuracy.

## Results

The literature search identified 10 studies of between 200 and 1,124 patients examining the association of acute breast radiation toxicity with predictor variables. Most studies reported acute skin toxicity scored according to RTOG, while only two studies used the CTCAE erythema scale (16, 25). Depending on the published study, the variables associated significantly with toxicity on multivariate analysis were: age (50 and over, dichotomized), body mass index (BMI), breast size or volume, fractionation schedule (hypo- vs. conventional fractionation, dichotomized), use of boost, smoking (ever smoked), and tamoxifen use (see Table 3). Chemotherapy showed significant effects but in opposite directions in two studies (19, 46) and was excluded. Interestingly, breast dose was not assessed as a continuous variable in any publication. Hypertension and diabetes were not significant on multivariate analysis in any study. One study used ordinal regression and did not report odds ratios, as endpoints were not dichotomized (20).

**Table 3:** Odds ratios (confidence intervals) of clinical predictor variables for acute breast toxicity reported from multivariate regression in previously published studies with significant associations in bold (BMI, body mass index; fractionation, fractionation treatment schedule).

**References**	**Twardella et al. (16)**	**Back (17)**	**Deantonio et al. (18)**	**Barnett et al. (19)**	**Terrazzino et al. (21)**	**Sharp et al. (22)**	**Tortorelli et al. (23)**	**Ciammella et al. (24)**	**De Langhe et al. (25)**
***N***	**478**	**234**	**155**	**1124**	**286**	**390**	**200**	**212**	**377**
Proportion acute breast toxicity^*^	**17.6%**	**31.4%**	**34.8%**	**36.5%**	**31.1%**	**21.3%**	**31.9%**	**15.0%**	**58.0%**
Age (per year) Age ≥50	0.98 (0.96–1.01)	Not significant^**^				**2.20 (1.00–4.80)**	0.97 (0.94–1.01)	Not significant	
BMI (per kg/m^2^) BMI ≥25 BMI ≥30	**1.09 (1.05–1.13)**	**2.10 (1.00–4.60)**			1.00 (0.92–1.09)	1.10 (0.60–2.10) **4.20 (2.10–8.30)**			**1.09 (not given)**
Breast volume (per liter) Breast volume > median Breast size > median Breast diameter Breast cup size ≥D		**3.60 (1.60–8.10)**		**2.09 (1.60–2.72)**	**1.14 (1.00–1.29)**		1.00 (1.00–1.01)	**2.47 (1.98–6.22)**	**2.83 (not given)**
Fractionation Hypo- vs. conventional			**0.45 (0.23–0.93)**						**0.08 (not given)**
Conventional vs. hypo-						**1.90 (1.00–3.50)**	**2.05 (1.00–4.20)**		
Boost use		Not significant			**4.90 (1.46–16.48)**			0.99 (0.98–1.00)	
Hypertension								Not significant	
Diabetes					Not significant			Not significant	
Smoking	0.86 (0.44–1.70)				Not significant	**2.50 (1.10–5.70)**			**2.71 (not given)**
Postop infection		Not significant		**1.49 (1.08–2.06)**				**3.46 (1.49–8.02)**	
Chemotherapy		Not significant	Not significant	**0.58 (0.41–0.82)**	Not significant	**1.80 (1.01–3.33)**	1.14 (0.53–2.43)	Not significant	0.95 (not given)
Tamoxifen use	1.54 (0.73–3.09)			**1.23 (1.07–1.41)**	Not significant	1.20 (0.70–2.10)	1.23 (0.55–2.77)		

* *The proportion of patients with acute breast toxicity is shown according to each study's endpoint definition*.

** *These studies only published p-values but no odds ratios for non-significant associations*.

### Predictive Clinical Model Development and External Validation

The distribution of patients across the three RGC development cohorts and the REQUITE validation cohort according to endpoint is shown in Table 4. Across the RGC cohorts (*n* = 2.031), there were 914 events of acute erythema (grade ≥2, 45.0%) and 175 events of acute desquamation (8.7%). It was noted that the incidence of desquamation was lower in the Cambridge IMRT cohort. In the REQUITE validation cohort, there were 1,969 and 2,057 patient datasets available for the endpoints acute erythema and acute desquamation, respectively. There were 450 patients with acute erythema (grade ≥2 erythema, 22.9%), and 192 patients with acute desquamation (grade 1≥ ulceration or grade ≥3 erythema, 9.3%).

**Table 4:** Number of patients by acute skin toxicity in the three RGC derivation cohorts and the REQUITE validation cohort; number and proportion of cases at the end of radiotherapy.

	**LeND**	**ISE**	**Cambridge**	**REQUITE**
**Eligible patients**	**409**	**478**	**1144**	**2057**
Acute erythema with RTOG or CTCAE grade ≥2	111 (27.1%)	358 (74.9%)	445 (38.9%)	450 (22.9%)
Acute desquamation with RTOG grade ≥2b or CTCAE grade ≥2c radiation dermatitis or grade ≥1 ulceration	62 (15.2%)	86 (18.0%)	27 (2.4%)	192 (9.3%)

Further detail regarding the distribution of clinical predictors in each cohort is available in Table 1. Median patient age in the REQUITE breast cohort was 58 years (range 23–80 years), similar to the RGC cohorts. REQUITE patients were treated with a median dose to the breast of 50 Gy (28.5–56 Gy) in 25 fractions (5–31), which is similar to the LeND and ISE cohorts. Patients in the Cambridge IMRT trial were exclusively treated with 40 Gy in 15 fractions. There was variation in use of boost between the different development cohorts and within the REQUITE multi-center cohort (see Methods). Although most other co-morbidities and co-medications were similarly distributed, the proportion of smokers was higher in the non-UK cohorts, whereas the proportion of overweight patients (BMI ≥25) was higher in the UK cohorts.

Final logistic regression models for both toxicity endpoints following backwards elimination are shown in Table 5. At this stage, variables that satisfied the *p* < 0.1 stepwise inclusion threshold in the development cohorts for both endpoints were BMI, breast size, hypo-fractionation, use of boost, and smoking status. Tamoxifen use was associated with acute erythema only (OR 1.25, CI 1.05–1.26; Table 5). Age 50 and over was eliminated from both models, acute erythema (OR 1.17, 0.89–1.53, *p* = 0.253) and acute desquamation (OR 1.45, 0.83–2.53, *p* = 0.194).

**Table 5:** Final logistic regression models (GLMM) following backwards elimination (*p* < 0.1) for acute breast toxicity endpoints in the RGC development cohorts.

	**Acute erythema**				**Acute desquamation**			
**Predictor**	**OR**	**CI lower**	**CI upper**	***p*-value**	**OR**	**CI lower**	**CI upper**	***p*-value**
BMI (per kg/m^2^)	1.05	1.02	1.08	<0.001	1.11	1.05	1.18	<0.001
Breast size (per sister size)	1.10	1.04	1.17	0.001	1.26	1.12	1.41	<0.001
Hypo-fractionation	0.22	0.05	0.89	0.033	0.08	0.05	0.13	<0.001
Boost use	1.34	1.06	1.69	0.013	1.79	1.24	2.57	0.002
Smoking (ever)	1.35	1.06	1.72	0.016	1.52	1.04	2.22	0.032
Tamoxifen use	1.25	1.01	1.56	0.044				

**Figure 1:**
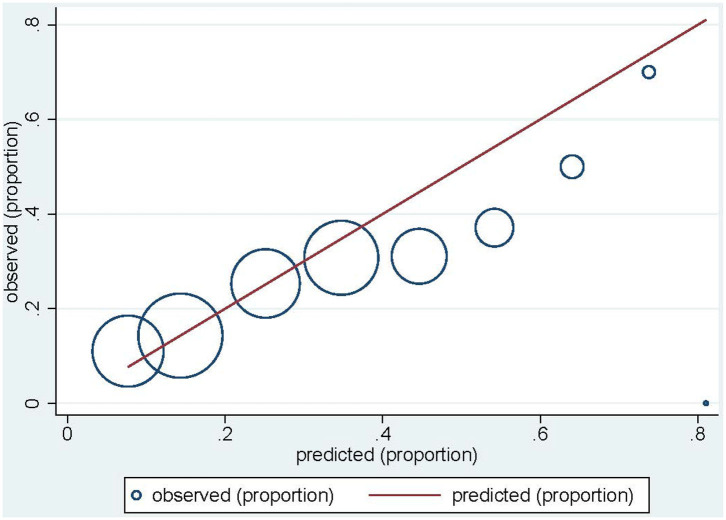
Calibration plot for acute erythema in the REQUITE validation cohort. Circles indicate the observed proportion of acute erythema per tenth of predicted probability. The red line indicates ideal calibration with a slope of 1.

Table 6 shows apparent, optimism-corrected (after bootstrapping) and validation performance of both risk prediction models. After correcting for optimism, the final model for acute erythema discriminated patients with and without grade ≥2 erythema undergoing EBRT following BCS with an AUC of 0.645 (CI 0.619–0.667). Agreement between observed and predicted proportions was seen with a calibration slope of 1.0319. The final log odds of acute erythema could be calculated as −2.265 + 0.049*BMI + 0.1*breast_size – 1.565*hypo-fractionation + 0.302*boost + 0.308*smoking + 0.234*tamoxifen. After re-calibrating the intercept, applying the final model to the REQUITE cohort gave a c-statistic (AUC) of 0.651 (CI 0.622–0.680), indicating the model performed equally well on validation, albeit with moderate calibration (slope = 0.665, 0.509–0.821) and a Brier score of 0.172 (Table 6). The calibration plot demonstrates that the model slightly over-predicts the probability of acute erythema in the REQUITE validation cohort (Figure 1), with a mean predicted probability of 25.7% against an observed incidence of 22.8%.

**Table 6:** Performance statistics for both predictive models in the RGC development cohorts and external validation performance in REQUITE.

	**Acute erythema**				**Acute desquamation**			
**Performance**	**Apparent**	**Average optimism**	**Optimism-corrected**	**External validation (REQUITE)**	**Apparent**	**Average optimism**	**Optimism-corrected**	**External validation (REQUITE)**
c-statistic (AUC)	0.644	−0.001	0.645	0.651	0.845	−0.002	0.849	0.697
Calibration slope	1.082	−0.050	1.032	0.665	1.067	−0.024	1.043	0.376
Brier score				0.172				0.085

**Figure 2:**
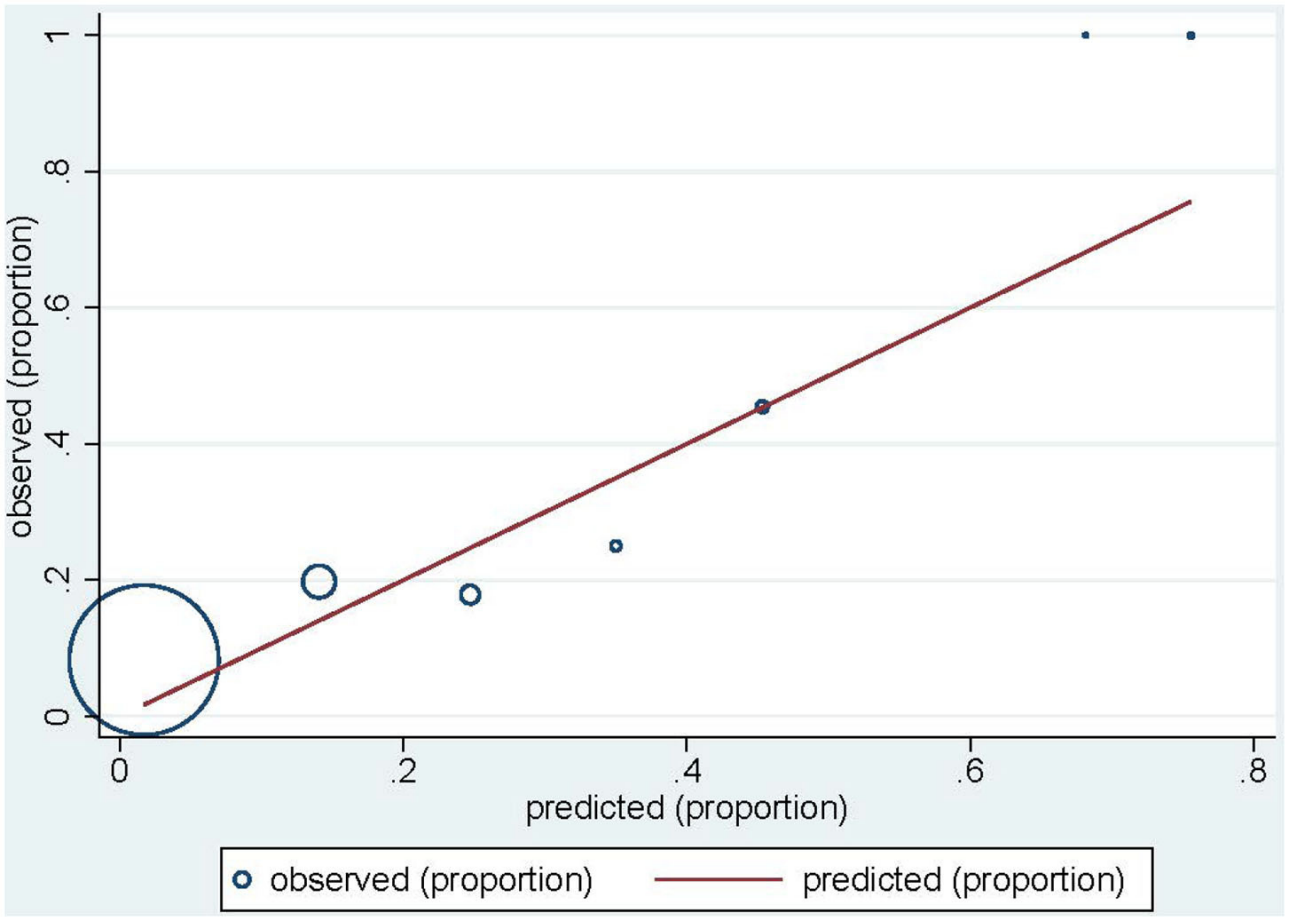
Calibration plot for acute desquamation in the REQUITE validation cohort. Circles indicate the observed proportion of acute desquamation per tenth of predicted probability. The red line indicates ideal calibration with a slope of 1.

The final model for acute desquamation developed in the joint RGC cohorts was able to discriminate patients with an optimism-corrected AUC of 0.847 (CI 0.817–0.873) and a calibration slope of 1.043. The log odds of acute desquamation could be calculated as −7.226 + 0.111*BMI + 0.240*breast_size – 2.592*hypo-fractionation + 0.606*boost + 0.435*smoking. Applying the final model to the REQUITE validation cohort with re-calibrated intercept, gave a c-statistic (AUC) of 0.697 (CI 0.658–0.737). This drop in AUC indicates relatively poorer discrimination performance, with equally poorer calibration (slope = 0.376, 0.260–0.492) (Table 6). The model significantly under-predicts the probability of acute desquamation in the REQUITE cohort, with a mean predicted probability of 3.0% against and observed incidence of 9.3% (Figure 2). The Brier score was 0.085.

## Discussion

The aim of this study was to develop and validate predictive models for acute skin erythema and acute desquamation following whole-breast external beam radiotherapy and breast-conserving surgery for breast cancer, which could be used without the need for detailed radiation dosimetry, in order to allow clinicians to estimate toxicity risk at the time of breast cancer diagnosis and before any treatment is planned. Previous work in prostate cancer showed that dosimetric models for radiation toxicity can be improved by adding clinical and co-treatment risk factors (14, 15).

The initial literature search of published predictors significantly associated with acute breast radiation toxicity in multivariate analysis confirmed a number of variables including BMI, breast size or volume, hypo-fractionation (protective), boost and tamoxifen use, and smoking status. Variables relating to BMI and breast size or volume have been most frequently reported in previous smaller cohorts (Table 3) as well as published randomized clinical trials (19, 47). Moreover, both aforementioned trials highlighted breast volume as a stand-alone predictor of acute radiation toxicity independent of dose inhomogeneity. Interestingly, none of the previous publications assessed breast dose as predictor in itself, only fractionation schedule. However, findings from the UK breast hypo-fractionation trials and radiobiology have shown that acute toxicity is related to total breast dose (48), not dose per fraction as for late toxicity (7). The protective association with hypo-fractionation reported in the literature is therefore likely due to the reduction in total dose for safe hypo-fractionation. Results of the literature search did not confirm an association with acute breast radiation toxicity for the predictors diabetes, cardiovascular disease and hypertension, whereas in the past radiation sensitivity has at least in part been attributed to the presence of cardiovascular disease or diabetes mellitus, which affects the microvasculature (49). However, it is likely that many patients enrolled in the reported cohorts were also on some form of anti-diabetic agent or a statin. Radioprotective effects of both metformin and gliclazide on human cells have been reported at least *in vitro* (50, 51), and there is evidence that statins may accelerate DNA repair (52) and reduce the expression of pro-inflammatory cytokines (53).

Although several studies have investigated the association of acute breast toxicity with clinical and treatment factors, to date, none have produced a clinical prediction model. Population-based measures of toxicity risk may not accurately reflect risk for an individual patient, but accurate prediction models can inform patients and clinicians about the future course of their condition or illness, thereby helping guide decisions about treatment. For a prediction model to be valuable, it should not only have predictive ability in the development cohort but must also perform well in a validation cohort. In the present study, the model to predict the risk of acute erythema following breast radiotherapy across RGC cohorts performed moderately well in the RGC cohorts and equally in the external REQUITE validation cohort with an AUC of 0.65, while calibration showed moderate agreement between predicted and observed toxicity outcomes in the validation cohort. On the other hand, performance of the model to predict the risk of acute desquamation following breast radiotherapy decreased relatively more in the external validation cohort (AUC = 0.70) than expected from internal validation (optimism-corrected AUC = 0.85), with relatively poor calibration.

Reasons why a predictive model may perform substantially differently between development and validation cohorts include over-fitting, missing important predictor variables, measurement errors of predictors, or differences in the patient cohort case mix. Measurement errors can arise from inter-observer variability across different cohorts and centers as well as use of different scales and time points to assess acute toxicity endpoints. Acute toxicity in both the Cambridge IMRT trial and the REQUITE study was assessed in the final week of radiotherapy. The acute reaction may not peak until 1–2 weeks after the end of treatment and hence could have been missed in some patients, although this would not have been the case in the ISE cohort study in which patients were assessed at the end and 6 weeks after the end of treatment. In the LeND study, acute toxicity was coded retrospectively from the medical notes, which may have led to bias if the original documentation was unclear. In both LeND and the Cambridge IMRT cohorts, toxicity was assessed using the RTOG scale, which does not separate out patients with oedema and might in part explain the lower proportion of cases with grade ≥2 erythema in these cohorts compared to the ISE cohort, although the proportion of cases in the REQUITE validation cohort is more similar to that of the LeND and Cambridge cohorts. The ability to detect and grade skin changes is also dependent on skin tone, which is not readily captured in the RTOG and CTCAE scales.

The model developed across the three RGC cohorts in this study included clinically relevant predictors which satisfied the relatively loose criteria for inclusion in the model (*p* < 0.1). The purpose of multivariate prediction modeling is estimation rather than testing for association with risk factors, and it may therefore be reasonable to include clinical predictors despite non-significant association or collinearity, to ensure that important predictors are not missed (54). In order to address over-fitting and to correct for optimism, bootstrapping was used as internal validation technique, but other studies with reasonably large datasets have used split-sample training-validation or cross-validation (55). It is possible but not very likely that a different internal validation method may have produced different results to the bootstrapping method used in the present study.

The value of the c-statistic (AUC) depends not only on the model of interest but also the even distribution of predictor and endpoint variables within a given patient population. Many radiotherapy patients present with a similar constellation of demographics and co-morbidities. They are also treated with similar plans, making discrimination a difficult task. In this study, acute desquamation was a relatively rare event in both the development and validation cohorts (8.7 and 9.3%), while the distribution of acute erythema within each dataset was somewhat more balanced toward cases (45.0 and 22.9%).

The distribution of clinical predictors between cohorts was broadly similar between the three development and validation cohorts, apart from smoking status and BMI, and none of the patients in the ISE cohort received chemotherapy. Overall, the distribution of clinical predictors was also similar to other previously published cohorts (21, 24, 25). Nevertheless, there was considerable heterogeneity between the centers within REQUITE with regards to treatment variables, such as dose fractionation, use of boost, and inclusion of patients who received prior adjuvant chemotherapy, as well as observed toxicity frequencies (39). Differences in radiotherapy techniques over time may have also affected generalizability of the prediction models to the validation cohort, as the patients in the three RGC development cohorts were on average treated more than 10 years before those enrolled in REQUITE. Certainly, there has been widespread update of intensity-modulated radiotherapy (IMRT) over that time, with almost 50% of patients enrolled in REQUITE treated in this way, whereas only some patients in the Cambridge trial cohort were randomized to IMRT and none of the patients in LeND and ISE received IMRT. Because of this and lack of data from previous literature, radiotherapy technique, such as IMRT, was not included in the model development phase.

A mixed modeling (GLMM) approach was chosen in this study to try and address issues of cohort heterogeneity and to relax the assumptions of independence of predictor variables. Using an alternative statistical method such as Lasso techniques, or data mining such as machine learning algorithms, may have identified other predictor variables or potential interactions in patients with several marginal risk factors (56). Machine learning algorithms are used with increasing frequency, in particular in the context of multi-dimensional “big data” such as electronic health records and radiotherapy imaging (57). However, the data available for this study, in particular from the slightly older RGC cohorts, were somewhat more limited and did not reach the multi-dimensionality usually associated with machine learning projects.

Despite these limitations, it is important to note that validation of the predictive model for acute erythema was achieved in the absence of detailed radiation dosimetry and notwithstanding the differences in radiotherapy techniques between treatment centers and countries particularly in the REQUITE cohort. The performance of this model across different cohorts in this study suggests that these findings are reproducible and generalizable beyond that of the original development dataset, whilst acknowledging the tendency for the model to over-predict in the external REQUITE cohort. The calibration plot demonstrates that the model can successfully identify high-risk patients and observed vs. expected rates were still correlated. This suggests that the model for acute erythema will simply benefit from further re-calibration of certain variable coefficients without redesigning the model from scratch. In the case of acute desquamation, further improvements using shrinkage and re-calibration would not affect the model's reduced discriminatory power in the validation cohort. To improve discrimination, the model would need to be revised, for example, by additional adjustment to regression coefficients of predictors with different strength or direction of effect in the RGC development compared to the REQUITE cohorts, stepwise selection of additional predictors, such as those relating to radiotherapy technique (e.g., IMRT), or re-estimation of all regression coefficients in the validation population. These approaches to update the model need to be balanced against the fact that the information in the original model would be neglected and would require further validation elsewhere.

To increase clinical relevance, novel performance measures such as net re-classification improvement (NRI) and net benefit (NB) could also be considered (58). Risk models without recommending clinical decisions are less likely to change treatment decision-making behavior than those that translate risk into a treatment decision recommendation (59). Nevertheless, the clinical risk model presented here without detailed radiation dosimetry can be used in practice relatively simply to predict a patient's probability of acute skin radiation toxicity at the time of breast cancer diagnosis, which can then be taken into account when discussion various treatment options with patients.

## Conclusions

While most published prediction research in the field of local breast cancer treatment toxicity continues to focus solely on model development, this study reports development and external validation of a predictive model for acute erythema following radiotherapy after breast-conserving surgery, which retained its moderate discriminatory power but will benefit from further re-calibration. A similar model to predict acute desquamation using clinical risk factors failed to validate in the REQUITE cohort. While other statistical or machine learning techniques may improve the performance of clinical risk models in the future, more accurate predictions are expected through the addition of genetic markers. This information could be considered when discussing breast cancer treatment options at the outset in particular with patients predicted at high risk of radiation toxicity.

## Data Availability Statement

The raw data supporting the conclusions of this article will be made available by the authors, without undue reservation.

## Ethics Statement

The studies involving human participants were reviewed and approved by Manchester North West UK NRES Approval 14/NW/0035. The patients/participants provided their written informed consent to participate in this study.

## Author Contributions

TRat conceived the study design and wrote the first draft of the paper. TRat and PS analyzed the data. TRat, PS, JC-C, TRan, RS, CW, and CT contributed to the interpretation of the data. CW is lead chief investigator and CT is deputy lead of the REQUITE study. JC-C is chief investigator of the ISE study, CEC is chief investigator of the Cambridge IMRT trial. RS is chief investigator of the LeND study. TRat, MA-B, MA, DA, GB, RB, JC-C, CEC, M-PF, KJ, GP, TRan, VR, BR, DD, MdS, ES, RS, BT-V, RV, and LV contributed patients to the participating studies. HS is the breast cancer patient advocate on the REQUITE study. AM and AW curated the database for the REQUITE study. All authors commented on and approved the final manuscript.

## Conflict of Interest

DA has been involved in the creation of the start-up NovaGray in 2015. DD is on the advisory board of Astra Zeneca, Bristol-Myers-Squibb, Roche/Genentech, Merck/Pfizer, Celgene, Noxxon, Mologen and has received investigator initiated grants from Bristol-Myers-Squibb, Boehringer Ingelheim, and Astra-Zeneca. ES received General speakers bureau Zeiss Meditec, travel support Zeiss Meditec. The remaining authors declare that the research was conducted in the absence of any commercial or financial relationships that could be construed as a potential conflict of interest.

## REQUITE Consortium Collaborators

Yolande Lievens^9^, Marc van Eijkeren^9^, Piet Ost^9^, Valérie Fonteyne^9^, Christel Monten^9^, Wilfried De Neve^9^, Stephanie Peeters^18^, Karin Haustermans^18^, Caroline Weltens^18^, Gilles Defraene^18^, Maarten Lambrecht^18^, Erik van Limberghen^18^, Erik Briers^25^, Celine Bourgier^6^, Muriel Brengues^6^, Roxana Draghici^6^, Francoise Bons^13^, Thomas Blaschke^26^, Christian Weiß^27^, Irmgard Helmbold^2^, Christian Weißenberger^28^, Petra Stegmaier^28^, Johannes Claßen^29^, Ulrich Giesche^29^, Marie-Luise Sautter-Bihl^30^, Burkhard Neu^30^, Thomas Schnabel^31^, Michael Ehmann^20^, Benjamin Gauter-Fleckenstein^20^, Carsten Herskind^20^, Marlon Veldwijk^20^, Jörg Schäfer^32^, Tommaso Giandini^33^, Marzia Franceschini^34^, Claudia Sangalli^19^, Barbara Avuzzi^19^, Sara Morlino^19^, Laura Lozza^19^, Gabriele Pietro^35^, Elena Delmastro^35^, Elisabetta Garibaldi^35^, Alessandro Cicchetti^15^, Ben Vanneste^17^, Bibiana Piqué-Leiva^5^, Meritxel Molla^5^, Alexandra Giraldo^5^, Monica Ramos^5^, Ramon Lobato-Busto^36^, Paloma Sosa-Fajardo^4,22^, Laura Torrado Moya^4,22^, Isabel Dominguez-Rios^22^, Irene Fajardo-Paneque^22^, Patricia Calvo-Crespo^4,22^, Ana Carballo^4,22^, Paula Peleteiro^4,22^, Olivia-Fuentes-Rios^3,4^, Antonio Gomez-Caamano^4,22^, Victoria Harrop^37^, Debbie Payne^38^, Manjusha Keni^39^, Samuel Lavers^23^, Simon Wright^40^, Sridhar Thiagarajan^40^, Luis Aznar-Garcia^40^, Kiran Kancherla^40^, Christopher Kent^40^, Subramaniam Vasanthan^40^, Donna Appleton^41^, Monika Kaushik^41^, Frances Kenny^41^, Hazem Khout^41^, Jaroslaw Krupa^41^, Kelly V. Lambert^41^, Simon Pilgrim^41^, Sheila Shokuhi^41^, Kalliope Valassiadou^41^, Ion Bioangiu^40^, Kufre Sampson^40^, Ahmed Osman^40^, Corinne Faivre-Finn^42^, Karen Foweraker^43^, Abigail Pascoe^43^, Claire P. Esler^43^, Tim Ward^44^, Daniel S. Higginson^45^, Richard G. Stock^46^, Sheryl Green^46^

^25^Patient advocate, Hasselt, Belgium

^26^Praxis für Strahlentherapie, Baden-Baden, Germany

^27^Klinikum Darmstadt, Institut für Radionkologie und Strahlentherapie, Darmstadt, Germany

^28^Zentrum für Strahlentherapie, Karlsruhe, Germany

^29^Klinik für Strahlentherapie, St. Vincentius-Kliniken gAG, Karlsruhe, Germany

^30^Klinik für Radioonkologie und Strahlentherapie, Städtisches Klinikum, Karlsruhe, Germany

^31^Klinik für Strahlentherapie und Radiologische Onkologie, Klinikum der Stadt Ludwigshafen gGmbH, Ludwigshafen, Germany

^32^Strahlentherapie Speyer, Germany

^33^Department of Medical Physics, Fondazione IRCCS Istituto Nazionale dei Tumori, Milan, Italy

^34^Department of Radiotherapy 2, Fondazione IRCCS Istituto Nazionale dei Tumori, Milan, Italy

^35^Department of Radiation Oncology, Fondazione del Piemonte per l'Oncologia Candiolo Cancer Institute, Candiolo (TO), Italy

^36^Department of Medical Physics, Complexo Hospitalario Universitario de Santiago, SERGAS, Santiago de Compostela, Spain

^37^Queen Elizabeth Hospital, University Hospitals Birmingham NHS Trust, Birmingham, United Kingdom

^38^Centre for Integrated Genomic Medical Research (CIGMR), Manchester, United Kingdom

^39^Department of Oncology, Derby Teaching Hospitals NHS Foundation Trust, Derby, United Kingdom

^40^Department of Oncology, Leicester Royal Infirmary, University Hospitals of Leicester NHS Trust, Leicester, United Kingdom

^41^Department of Breast Surgery, Glenfield Hospital, University Hospitals of Leicester NHS Trust, Leicester, United Kingdom

^42^Division of Cancer Sciences, University of Manchester, Manchester, United Kingdom

^43^City Hospital, Nottingham University Hospitals NHS Trust, Nottingham, United Kingdom

^44^Patient advocate, Pelvic Radiation Disease Association, United Kingdom

^45^Department of Radiation Oncology, Memorial Sloan Kettering Cancer Center, New York, NY, United States

^46^Department of Radiation Oncology, The Icahn School of Medicine at Mount Sinai, New York, NY, United States
